# Laparoscopic liver resection for pediatric liver tumors: a systematic review and meta-analysis

**DOI:** 10.3389/fped.2026.1756373

**Published:** 2026-07-24

**Authors:** Hanlei Yan, Baijun Zheng, Donghao Tian, Ya Gao

**Affiliations:** 1 Department of Pediatric Surgery, The Second Affiliated Hospital, Xi’an Jiaotong University, Xi’an, China; 2 Institute of Neurobiology, Environment and Genes Related to Diseases Key Laboratory of Chinese Ministry of Education, Xi'an Jiaotong University, Xi’an, China

**Keywords:** hepatectomy, laparoscopic, liver tumor, pediatric, systematic review and meta-analysis

## Abstract

**Purpose:**

To evaluate the safety and feasibility of laparoscopic liver resection (LLR) for pediatric liver tumors.

**Methods:**

We systematically searched PubMed, Web of Science, Cochrane Library, Embase, and Chinese databases (CNKI, WanFang, VIP) for studies on LLR for pediatric liver tumors. Following PRISMA guidelines, nine studies (144 LLR patients) were selected and evaluated using the Methodological Index for Non-Randomized Studies (MINORS). Meta-analysis of single-arm proportions and continuous variables was conducted using R 4.4.1, with comparative analysis against open liver resection (OLR).

**Results:**

The pooled mean maximum tumor diameter was 5.69 cm (95% CI: 4.85–6.53). Mean operative time was 208.77 min (95% CI: 153.74–263.80), and intraoperative blood loss 88.80 mL (95% CI: 31.33–146.27). Transfusion proportion was 32% (95% CI: 16%–54%), hospital stay was 6.8 days (95% CI: 4–9.6), and the overall postoperative complications 16% (95% CI: 10%–24%) including 11% bile leakage (95% CI: 6%–19%). Notably, no gas embolism or port site metastasis occurred. The hepatoblastoma recurrence proportion was 9% (95% CI: 5%–19%). LLR required significantly longer operative time (+60.02 min; *p* < 0.0001) but achieved significantly shorter hospitalization (−2.76 days; *p* < 0.0001) compared to OLR. Intraoperative blood loss did not differ significantly between groups (*p* = 0.7138).

**Conclusion:**

LLR appears safe and feasible for pediatric liver tumors in selected patients. Limited evidence and heterogeneous patient selection warrant propensity score matching and multicenter prospective studies to establish surgical indications and long-term oncologic outcomes.

## 1 Introduction

Liver tumors represent a relatively rare subset of pediatric malignancies, comprising approximately 1% to 4% of childhood cancers, with two-thirds classified as malignant (1). Liver partial resection remains a cornerstone of curative treatment. While laparoscopic liver resection (LLR) has been established in adult surgery since 1992 and is supported by high-level evidence demonstrating advantages in recovery and cost-effectiveness over open liver resection (OLR) (2–5), its application in pediatric oncology has evolved more gradually.

The pediatric experience with LLR began in 2006 with Yoon et al.'s first reported case of laparoscopic left lateral resection for benign tumors (6), followed by subsequent technical explorations such as Gao Ya's left hepatectomy (7). Over time, observational studies have documented the feasibility of various minimally invasive approaches in children, including anatomical resections (e.g., left lateral lobe, right hemiliver), non-anatomical segmental resections, ALPPS (associating liver partition and portal vein ligation for staged hepatectomy) and robot-assisted procedures (8–11).

Despite these advancements, the evidence base remains constrained by the rarity of pediatric liver tumors. Current literature predominantly consists of single-center studies with limited sample sizes, underscoring the need for comprehensive synthesis. This study systematically evaluates the clinical characteristics and outcomes of laparoscopic hepatectomy for pediatric liver tumors through meta-analysis, aiming to clarify its role in contemporary surgical practice.

## 2 Methodology

### 2.1 Literature search strategy

Two researchers independently performed literature searches from inception to December 12, 2024, using PubMed, Web of Science, Cochrane Library, Embase, CNKI, WanFang, and VIP datasets. The complete search strategy (subject terms and free words) and the full inclusion and exclusion criteria are available in Supplementary File 1. Our search strategy incorporated the following keywords: “ Pediatrics ”, “ Liver Neoplasms ”, “ Hepatoblastoma ”, “ Hepatectomy ”, “ Laparoscopes ”, “ Minimally Invasive Surgical Procedures ”, etc. The Chinese search terms were “pediatrics,” “liver tumor,” “hepatoblastoma,” “hepatectomy,” “laparoscopy,” and “minimally invasive.” The researchers compared the screening results, and sought the opinions of a third researcher to decide whether to include the literature with disagreements. The principles for screening literature were based on PRISMA (Preferred Reporting Items for Systematic Reviews and Meta-Analyses). This study has been registered with PROSPERO(CRD42024619850).

### 2.2 PICOS strategy

Participant/Population(s): pediatric patients (<18 years) with benign/malignant liver tumors;

Intervention(s): pediatric patients underwent laparoscopic hepatectomy for benign/malignant liver tumors;

Comparator(s)/Control: pediatric patients underwent open hepatectomy for benign/malignant liver tumors or no control group;

Outcome(s): Resection margin, length of hospital stay, postoperative complications including overall complications, bile leakage and others, follow-up time and survival outcomes;

Study: Randomized controlled trials (RCTs), prospective observational studies, retrospective cohort studies, and case series.

### 2.3 Data extraction

Two investigators independently extracted data using standardized forms, with discrepancies resolved through discussion or third-party adjudication. The extracted information included study characteristics (publication year, study design, country, and sample size), participant demographics (age, gender), and tumor features (size, location, stage, pathology). Surgical parameters encompassed procedure type, operation time, intraoperative blood transfusion volume, and intraoperative imaging (ultrasound/ICG). Postoperative outcomes focused on resection margin status, hospital stay duration, complications (overall/bile leakage), and survival outcomes with follow-up period.

### 2.4 Risk of bias assessment

Two researchers independently checked the quality of each study using the MINORS tool (12). MINORS is a 12-item checklist for evaluating non-randomized surgical studies. For studies without comparison groups, only the first 8 items were used. Any disagreements were discussed and resolved with a third researcher. Publication bias was planned to be assessed via funnel plot symmetry and Egger's regression test; however, these analyses were not performed due to the limited number of included studies (*n* < 10), following Cochrane recommendations for meta-analyses with small sample sizes (13).

### 2.5 Sensitivity analysis

To assess the robustness of our pooled estimates and potential influence of individual studies on the overall conclusions, we performed a leave-one-out sensitivity analysis. We systematically repeated the meta-analysis by sequentially excluding each included study, and examining whether the direction and magnitude of pooled estimates remained consistent. This approach was conducted as recommended by the Cochrane Handbook for Systematic Reviews of Interventions (14).

### 2.6 Statistical analysis

Data were analyzed using R software (version 4.4.1). Continuous variables are presented as median (range) or mea*n* ± standard deviation. For studies reporting medians, data were converted to means using established transformation methods (15–17). Heterogeneity was evaluated through I^2^ statistics and Cochran's *Q* test, with random-effects models employed when I^2^ > 50% and/or *p* ≤ 0.1. Statistical significance was set at *p* < 0.05.

## 3 Results

### 3.1 Literature search results and characteristics of included studies

**Figure 1:**
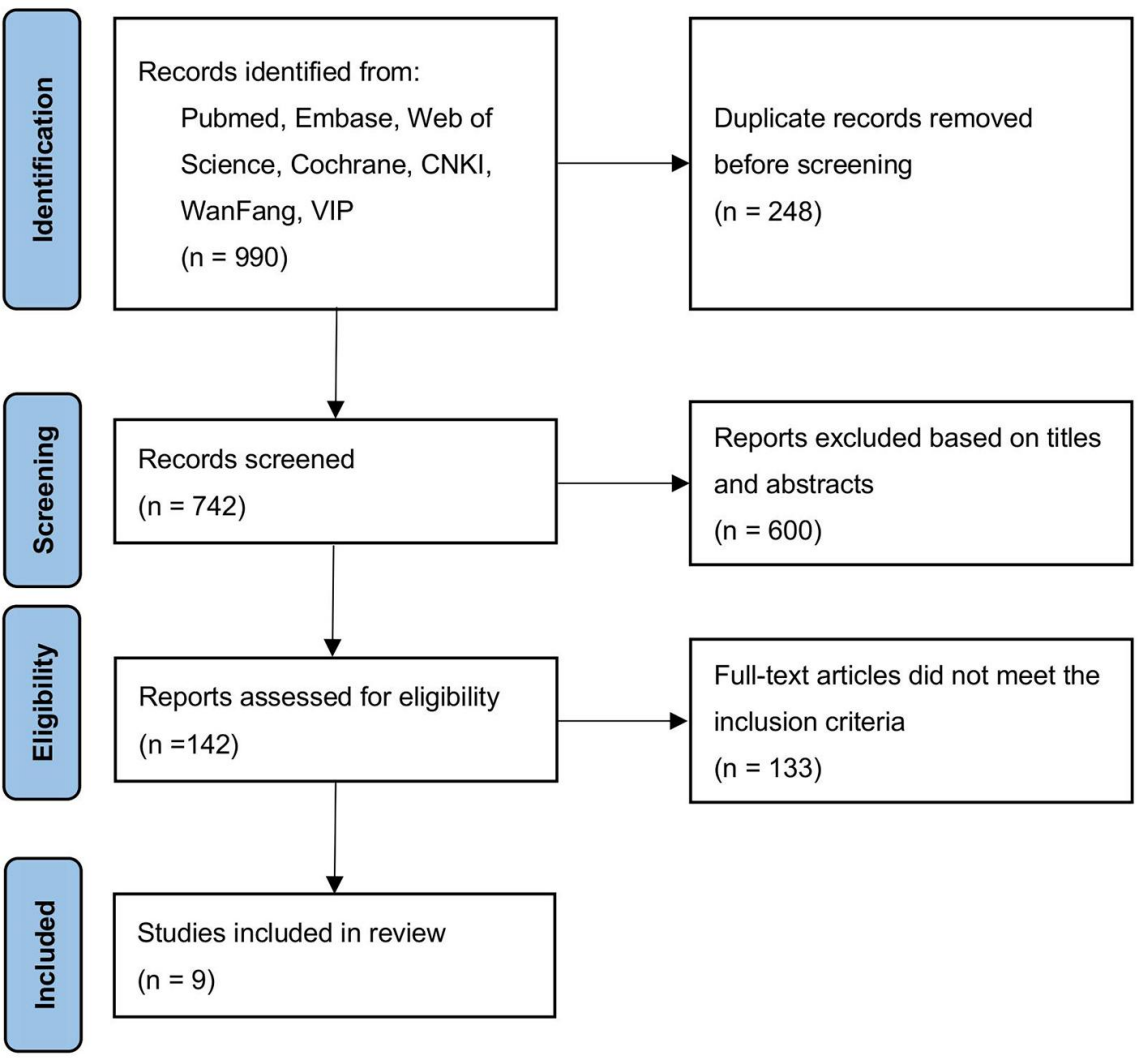
PRISMA flow diagram of study selection.

The literature screening process is summarized in Figure 1. From 990 initially identified articles, 142 full-text publications were assessed, ultimately including 9 studies (2016–2022) (9, 10, 18–24). These comprised 7 case series and 2 case-control studies. Key characteristics of the included studies are presented in Tables 1–3, covering demographics, perioperative, and survival outcomes.

**Table 1:** General characteristics of included studies.

Author, year	Study design	Nation	Age	Gender (male)	LLR	Diagnosis^a^	Major resection
Veenstra MA, 2016 (18)	CS	USA	2.7y (9m−17y)	17	36	20/36	≥20
Zheng B, 2019 (19)	CCS	China	22.3m ± 7.6m	10	LLR: 17	17/17	10/17
			24.7m ± 5.1m	14	OLR: 26	26/26	18/26
Kwon H, 2019 (9)	CS	South Korea	26 m (9d−15.4y)	10	19	13/19	5/19
Wang J, 2020 (20)	CCS	China	7.6m ± 3.8 (2y−13y)	15	LLR: 21	9/21	≥5
			7.0m ± 3.7 (2y−14y)	6	OLR: 9	7/9	≥3
Murawski M, 2021 (10)	CS	Poland	4m−16y	3	6	0/6	NR
Li Q, 2021 (21)	CS	China	7y−2y	4	6	3/6	5/6^b^
Zhou H, 2021 (22)	CS	China	66.26 m (6m−14y)	9	15	10/15	NR
Zheng B, 2022 (23)	CS	China	21.2 ± 9.01m	8	17	14/17	11/17
Qiu R, 2022 (24)	CS	China	13d−3y	6	7	7/7	5/7

CS, case series; CCS, case control study; LLR, laparoscopic liver resection; Major resection (25–27): including hemihepatectomy, trisegmentectomy, middle hepatectomy, and difficult liver segment (S4a, S7, and S8); "≥": limited to reported data due to incomplete surgical details in source materials.

a number of hepatoblastoma cases/total number of cases; b: including 1 case of laparoscopic caudate lobectomy. NR, Not Reported.

**Table 2:** Perioperative characteristics of LLR for pediatric liver tumors.

Author, year	Resection type	Tumor size (cm)	PRE/POST-TEXT Stage (n)	Operative time (min)	Blood loss (mL)	Blood transfusion	Intraoperative imaging	Length of hospital stay (days)	Resection edge (mm)	Complication (bile leak/all)
Veenstra MA, 2016 (18)	Segmentectomy	5 (2–16)	NR	74 (50–110)	10 (10)	1/11	Ultrasound	3 (2–6)	Negative	0/5
	Lobectomy	5 (2–16)		120 (48–200)	50 (10–5)	0/5		4 (2–5)		
	Hemihepatectomy	4 (2–9)		195 (55–450)	63 (10–250)	4/20		5 (2–9)		
Zheng B, 2019 (19)		7.0 ± 4.6 (3.6–16)	I/II/III/IV (2/5/9/1)^a^	246 ± 47.4	77.5 ± 39.2	9/17	Ultrasound	7.3 ± 1.3	Negative	3/4
Kwon H，2019 (9)		5.3 (2–8.1)	I/II/III/IV (4/6/3/0)^a^	230 (60–360)	3.8 (0.6–19.4 mL/kg)	9/19	ICG	7 (3–31)	7.5 (1–38)	0/0
Wang J，2020 (20)		7.2 ± 2.0 (4–10)	NR	182.3 ± 66.1 (60–300)	164.4 ± 107.4 (20–300)	102.2 ±100.2 (0–200 )	Ultrasound or ICG	6.2 ± 1.6 (5–10)	NR	0/0
Murawski M，2021 (10)		4.7 (2.2–7)	I/II/III/IV (5/1/0/0)^a^	177 (90–270)	4 cases ≤ 50; 300–400 per case; 800 one case	2/6	Ultrasound	4.8 (3–7)	Negative	0/0
Li Q, 2021 (21)		4.5 (3–7)	NR	90 ± 9 (80–100)	83 ± 26 (50–120)	0	NR	4 (3–5)	Negative	0/0
(22)		NR	NR	253.1 ± 41.5 （180–290）	50.76 ± 7.56	NR	NR	12.37 ± 1.76	NR	2/4
Zheng B, 2022 (23)		6.2 ± 3.44 (3.2–14.4)	I/II/III/IV (5/9/0/0)^b^	234.4 ± 56.22 (70–360)	60.7 (20–300)	NR	ICG	NR	Negative (1.2 ± 0.37)	1/1
Qiu R，2022 (24)		6.5 (2.5–8.7)	I/II/III/IV (2/5/0/0)^b^	359.3 ± 117.2 (250–570)	280 (50–600)	NR	ICG	NR	Negative	2/2

The numerical types in the table are median (range), mean ± standard deviation (range), number of events/total number of cases.

NR, Not Reported.

a PRETEXT stage.

b POST-TEXT stage.

**Table 3:** Survival outcomes after LLR for pediatric liver tumors.

Author, year	Follow-up period (months)	Survival status (n)
Veenstra MA, 2016 (18)	12 (6–36)	HB: lung recurrence (1)
Zheng B, 2019 (19)	2–43	HB: Relapse (3)
Kwon H, 2019 (9)	64 (18–156)	NB: Relapse (1)
Wang J, 2020 (20)	8–48	No recurrence
Murawski M, 2021 (10)	NR	NR
Li Q, 2021 (21)	2–23	No recurrence
Zhou H, 2021 (22)	NR	NR
Zheng B, 2022 (23)	18 (1–34)	HB: lung recurrence (1)
Qiu R, 2022 (24)	24 (9–37)	No recurrence

HB, hepatoblastoma; NB, neuroblastoma; NR, Not Reported.

A total of 179 pediatric patients with liver tumors from 9 included studies were analyzed, comprising 46 benign cases (26%) and 133 malignant cases (74%), among which 126 were hepatoblastoma (70%). Within the LLR group (144), there were 44 benign tumors (31%) and 100 malignant tumors (69%), including 93 cases of hepatoblastoma (65%). In the meta-analysis comparing LLR and OLR, 73 pediatric patients with liver tumors were included, consisting of 11 benign tumors (15%) and 62 malignant tumors (85%), with 59 cases of hepatoblastoma (81%).

The 9 articles included 35 cases of open liver resection and 144 cases of LLR. Among them, Veenstra MA et al. (18) reported 5 cases of hand Assist/hybrid liver resection. Wang J et al. (20) reported one case of hand-assisted laparoscopic hepatectomy; Kwon H et al. (9), Murawski M et al. (10) and Zheng B et al. (23) each reported one case of conversion to open surgery.

PRETEXT/POST-TEXT stage data were available in 5 studies. Among these, the majority of cases were classified as early stage: 23 cases were classified as PRETEXT I/II, and 21 cases were POST-TEXT I/II. Additionally, 3 cases of PRETEXT III were reported by Kwon H et al. (9), and Zheng B et al. (19) reported 9 cases of PRETEXT III and 1 case of PRETEXT IV.

### 3.2 Quality evaluation

Study quality was evaluated using the MINORS criteria (Table 4). For case series (maximum score 16), included studies scored 12 (range 10–14). Case-control studies (maximum score 24) achieved a uniform score of 19.

**Table 4:** Risk of bias assessment for included studies with MINORS.

MINORS Items	Veenstra MA, 2016 (18)	Zheng B, 2019 (19)	Kwon H, 2019 (9)	Wang J, 2020 (20)	Murawski M, 2021 (10)	Li Q, 2021 (21)	Zhou H, 2021 (22)	Zheng B, 2022 (23)	Qiu R, 2022 (24)
Clearly stated aim	2	2	2	2	2	2	2	2	2
Consecutive patients	2	2	2	2	2	2	2	2	2
Prospective collection of data	2	2	2	2	2	2	2	2	2
Endpoints appropriate to aim of study	2	2	2	2	2	2	2	2	2
Unbiased assessment of the study endpoint	0	0	2	0	0	0	0	0	0
Follow-up period appropriate to aim of study	2	2	2	2	2	0	2	2	2
Loss to follow up less than 5%	2	2	2	2	2	2	2	2	2
Prospective calculation of the study size	0	0	0	0	0	0	0	0	0
An adequate control group	/	2	/	2	/	/	/	/	/
Contemporary groups	/	2	/	2	/	/	/	/	/
Baseline equivalence of groups	/	2	/	2	/	/	/	/	/
Adequate statistical analyses	/	1	/	1	/	/	/	/	/
**Total**	**12/16**	**19/24**	**14/16**	**19/24**	**12/16**	**10/16**	**12/16**	**12/16**	**12/16**

Total indicates the achieved score out of the maximum possible score for the corresponding study type.

### 3.3 Meta-analysis results

**Figure 2:**
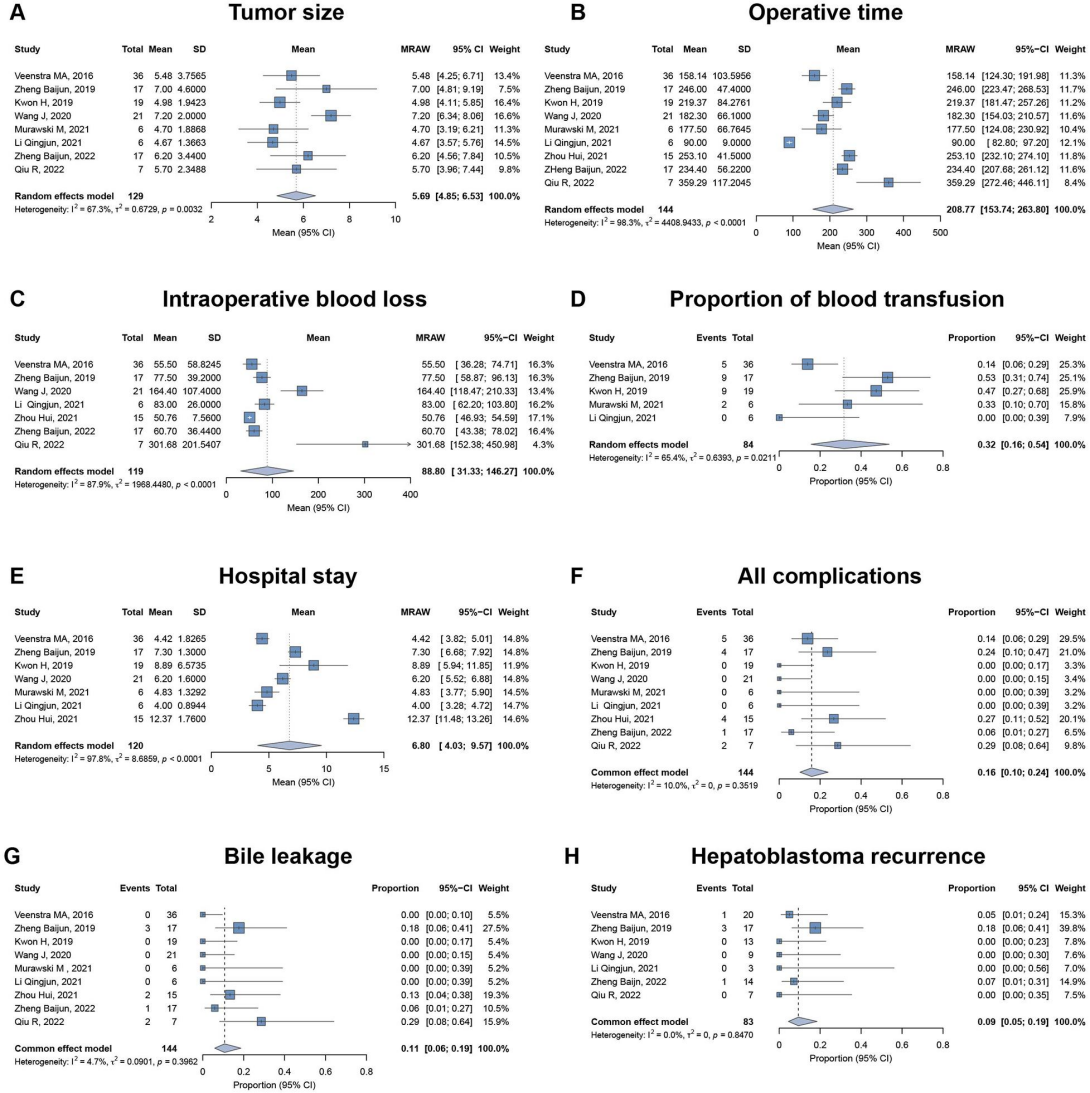
**(A)** Tumor size, **(B)** Operative time, **(C)** Intraoperative blood loss, **(D)** Proportion of blood transfusion, **(E)** Hospital stay, **(F)** All complications, **(G)** Bile leakage, and **(H)** Hepatoblastoma recurrence. Forest plot of clinical characteristics associated with LLR for pediatric liver tumors. Each blue square represents the raw mean (MRAW) or proportion from an individual study, with horizontal lines indicating 95% confidence intervals (CIs). The size of the square reflects the study's weight in the random/common effects meta-analysis. The diamond at the bottom shows the pooled mean or proportion and its 95% CI. Heterogeneity was assessed using Cochran's *Q* test (*p* value), the I^2^ statistic, and *τ*^2^, with random-effects models employed when I^2^ > 50% and/or *p* ≤ 0.1.

Pooled analysis of the clinical characteristics associated with LLR in pediatric patients demonstrated the following outcomes: the mean maximum tumor diameter was 5.69 cm (95% CI: 4.85–6.53), with a mean operative duration of 208.77 min (95% CI: 153.74–263.80). Intraoperative parameters revealed a mean blood loss of 88.80 mL (95% CI: 31.33–146.27), while 32% of cases (95% CI: 16%–54%) required perioperative blood transfusion (Figures 2A–D). Postoperative outcomes showed a mean hospital stay of 6.8 days (95% CI: 4–9.6) and an overall complications proportion of 16% (95% CI: 10%–24%). Procedure-specific complications included a 11% incidence of bile leakage (95% CI: 6%–19%), with HB demonstrating a 9% recurrence (95% CI: 5%–19%) (Figures 2E–H).

**Figure 3:**
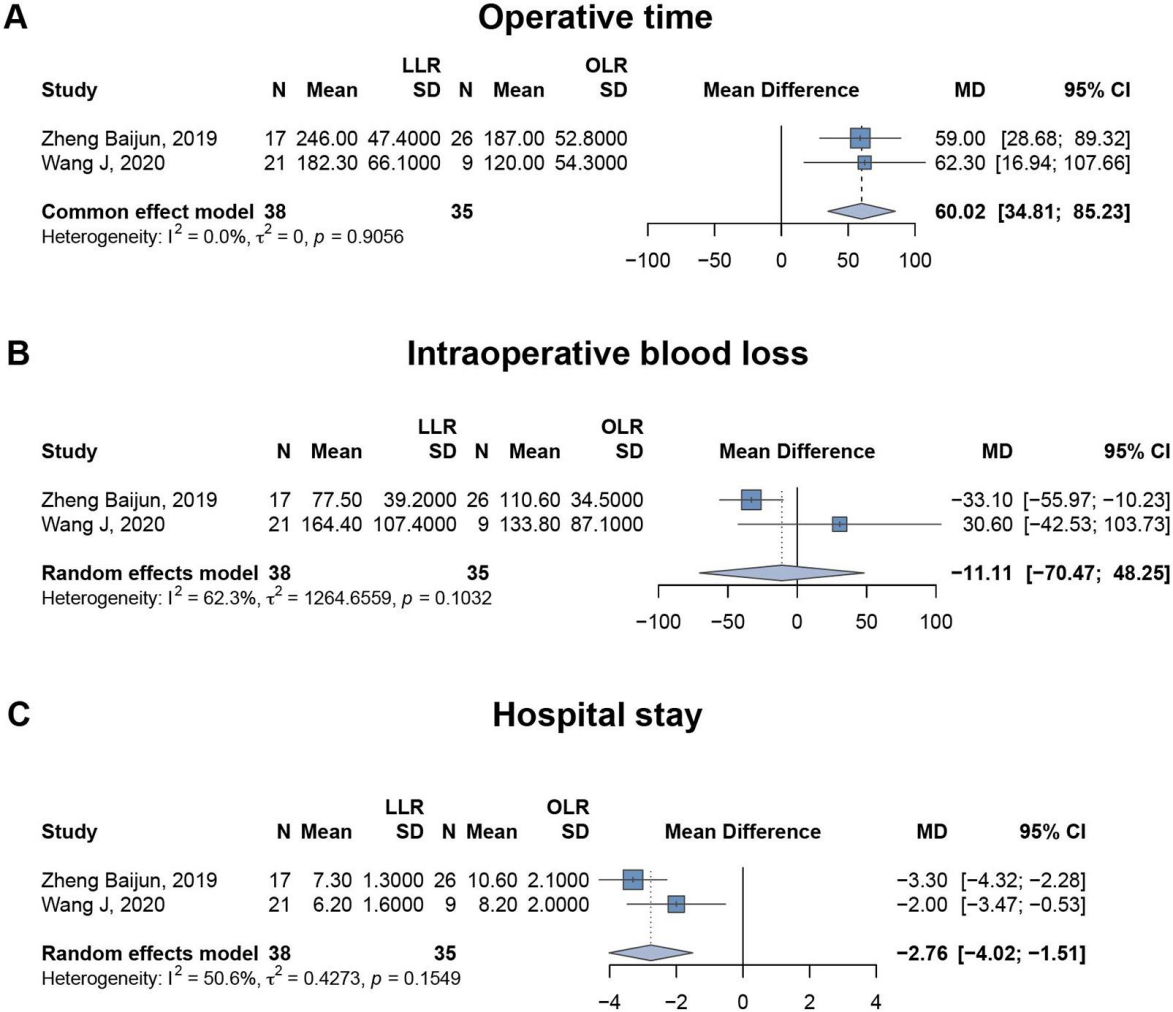
**(A)** Operative time, **(B)**, Intraoperative blood loss, **(C)**, Hospital stay. Forest plot comparing clinical characteristics between LLR and OLR for pediatric liver tumors. The sample size (N), mean, and standard deviation (SD) are presented for both the LLR and OLR groups. Each blue square represents the mean difference (MD) of an individual study, with the horizontal line indicating its 95% confidence interval (CI). The size of the square reflects the study's weight in the random/common effects meta-analysis. The diamond at the bottom shows the pooled MD and its 95% CI. The dashed vertical line (MD = 0) represents the line of no effect. Heterogeneity was assessed using Cochran's *Q* test (*P* value), the I^2^ statistic, and *τ*^2^, with random-effects models employed when I^2^ > 50% and/or *p* ≤ 0.1.

Two case control studies showed (Figure 3): LLR procedures required approximately 1 h longer operative time compared to OLR [mean difference (MD): +60.02 min; 95% CI: 34.81–85.23; z = 4.67; *p* < 0.0001], with no heterogeneity (I^2^ = 0%). For intraoperative blood loss, no statistically significant difference was observed between LLR and OLR (MD: −11.11 mL; 95% CI: −70.47–48.25; *p* = 0.7138), although moderate heterogeneity was present (I^2^ = 62.3%), likely reflecting differences in patient populations (Zheng B, 2019: 100% hepatoblastoma; Wang J, 2020: 53% hepatoblastoma). Regarding postoperative hospital stay, LLR was associated with a significantly shorter hospitalization (MD: −2.76 days; 95% CI: −4.02 to −1.51; z = −4.32; *p* < 0.0001), with I^2^ = 50.6%.

These findings should be interpreted with substantial caution. The evidence base comprises only two small case-controlled studies with a combined sample of 73 patients (LLR: 38, OLR: 35). Given the observational design and absence of confounding adjustment, these comparisons are descriptive rather than causal. Patient selection bias likely favors LLR in early-stage, anatomically favorable cases, which may explain the differences in operative time and hospital stay. Details of the two comparative studies are presented in Supplementary Table S1.

### 3.4 Sensitivity analysis

Pooled estimates remained consistent across all sensitivity scenarios for the majority of clinical characteristics and outcomes. Tumor size, all complications, bile leakage, and hepatoblastoma recurrence demonstrated maximal variation of approximately 1% across all exclusion scenarios, indicating highly stable findings. In contrast, operative time, intraoperative blood loss, hospital stay, and blood transfusion proportion showed modest variation ranging from 6.8% to 12%, which remained acceptable given the clinical heterogeneity of included studies (Supplementary Figure S1 and Supplementary Table S2). Exclusion of Wang J et al. (20) resulted in a reduction of pooled blood loss from 88.80 mL to 65.75 mL, reflecting the higher blood loss reported in that study (mean: 164.4 mL) compared to others. Similarly, exclusion of Zhou Hui (22) notably reduced the pooled hospital stay from 6.80 to 5.70 days, reflecting the longer length of stay (mean: 12.37 days) reported in that study. Conversely, exclusion of Veenstra MA et al. (18) increased the pooled transfusion proportion from 32% to 45%, consistent with the lower transfusion proportion (14%) reported in that study relative to the remaining studies. Nevertheless, confidence intervals remained overlapping across all exclusion scenarios and the direction of estimates was unchanged, supporting the overall robustness of the primary findings.

It is noteworthy that both Veenstra MA et al. (18) and Wang J et al. (20) included hand-assisted or hybrid laparoscopic procedures that retain a laparotomy incision, which are not pure laparoscopic resections. To account for this methodological concern, we performed a sensitivity analysis by simultaneously excluding both studies. (Supplementary Table S2A-H). Bidirectional effects were observed: tumor size and blood loss decreased, while operative time, transfusion, hospitalization, complications, and recurrence increased. Nevertheless, confidence intervals remained overlapping with pooled estimates from all studies, confirming robustness despite these directional variations.

## 4 Discussion

Surgical intervention remains the cornerstone of pediatric liver tumor management, encompassing three principal approaches: biopsy, partial hepatectomy, and liver transplantation. While OLR offers direct tactile feedback and enhanced hemorrhage control, the evolution of minimally invasive techniques—driven by advancements in surgical instrumentation, preoperative imaging, and patient demand for improved cosmesis and recovery—has established laparoscopy as a viable alternative (28). Our meta-analysis suggests that LLR appears safe and feasible in selected pediatric patients. However, current evidence remains limited by heterogeneity and small sample sizes. Multicenter prospective studies and propensity score-matched analyses are required to establish standardized surgical indications and to evaluate long-term outcomes.

Intraoperative hemorrhage, a critical challenge in hepatic surgery, averaged 89 mL in LLR cases. Comparative analyses revealed an 11 mL reduction in blood loss compared to OLR, aligning with adult studies (5). This benefit likely stems from pneumoperitoneum-induced hemostasis, hepatic inflow control, and low central venous pressure management. Notably, three cases (3/138, 2%) required conversion to laparotomy due to uncontrolled bleeding, underscoring the importance of timely open conversion when oncological safety is compromised.

LLR procedures averaged 3.5 h—1 h longer than OLR. This disparity reflects multifactorial influences: tumor size [maximum reported diameter: 16 cm (18, 23)], relationship with vessels, anatomical complexity，and surgical expertise. Currently, the minimum age for LLR surgery is 9 days old (9). Compared with other included literature, Qiu R et al. (24) reported longer operation time and more intraoperative blood loss, which was related to the fact that most of their cases (5/7) were “ major LLR ”. Despite prolonged surgery, LLR facilitated accelerated recovery, with a mean hospitalization of 6.8 days (3 days shorter than OLR) and earlier oral intake initiation (59/74 cases resumed feeding within 1–3 days). These advantages enable prompt postoperative chemotherapy initiation, critical for malignant cases. This benefit has been clearly demonstrated in adult studies (29).

The overall complication rate following LLR was approximately 16%, comparable to the wide range reported after OLR (16%–58%) (30–33). Bile leakage occurred in 11% of LLR cases (95% CI: 6%–19%), with only one patient (11% of those with bile leakage) requiring reoperation. In contrast, OLR bile leakage rates ranged from 3.4% to 12%, but reintervention rates varied substantially (0%–89%), reflecting heterogeneous management strategies across studies (30–33). Zheng B et al. (19) attributed decreased bile duct injuries in LLR to enhanced visualization of biliary anatomy under magnification, potentially mitigating intraoperative ductal trauma. Notably, no instances of gas embolism, port-site metastasis, hepatic failure, or perioperative mortality were reported in LLR.

Pediatric liver malignancies predominantly included hepatoblastoma, hepatocellular carcinoma, and sarcomas, while benign tumors comprised hemangiomas, hamartomas, and FNH. Benign lesions often warranted resection due to persistent symptoms, diagnostic uncertainty, or mass effects on adjacent organs (1, 10). Hepatoblastoma represents the most common pediatric malignant liver tumor. Among hepatoblastoma cases, the pooled recurrence rate was 9% (95% CI: 5%–19%), with all recurrence events localized to the lungs. Zheng B et al. (19) reported no statistically significant difference in 3-year event-free survival (EFS) between the LLR and OLR groups (75.0% vs. 76.9%), suggesting that LLR does not compromise oncological outcomes in selected hepatoblastoma patients. However, a formal meta-analysis of survival outcomes was not feasible due to substantial inter-study heterogeneity in tumor composition and the absence of histology-stratified survival data. Consequently, this single comparative study finding is insufficient to draw definitive conclusions and warrants cautious interpretation.

Notably, neoadjuvant chemotherapy enabled downstaging of PRETEXT II/III hepatoblastomas to expand LLR eligibility: Qiu et al. (23) reported downgrading 5 PRETEXT III cases to stage II and 1 stage II to I using the 2016 Chinese Children's Cancer Group regimen, while Kwon H et al. (9) achieved tumor size reduction from 9.9 cm (6.0–15.0) to 5.3 cm (2–8.1) with Children's Oncology Group regimen. Reflecting this selective application of LLR, stage analysis of included studies revealed that LLR was predominantly applied to PRETEXT/POST-TEXT I/II cases. The stratification by disease stage and tumor size represents key confounding factors that must be considered when interpreting the comparative outcomes between LLR and OLR.

While LLR is well-established in adult patients, its indications in pediatric patients remain unclear. Initial criteria from the 2008 Louisville Statements (25) restricted LLR to solitary lesions <5 cm in segments S2–6 (“laparoscopic sites”). Current indications have expanded beyond tumor location/size, facilitated by accumulated expertise, 3D preoperative imaging, advanced energy devices, intraoperative ultrasound, and ICG navigation. Major LLR accounted for 52% (60/123) of cases, though complex or giant tumors still require high-volume centers. Tumor size relative to pediatric anatomy complicates difficulty stratification; a Russian classification system (34) categorizes pediatric LLR complexity (low/medium/high) to predict complication risks.

## 5 Conclusion

LLR appears safe and feasible in selected pediatric patients, but current evidence is limited by heterogeneous patient populations with inconsistent selection criteria, and insufficient histology-stratified reporting. To mitigate selection bias, future studies should prioritize propensity score-matched (PSM) analyses. Furthermore, multicenter prospective registries (ChiCTR2400093933) with standardized, stratified outcome reporting are essential to definitively establish LLR indications and validate its safety and efficacy.

## 6 Limitation

This meta-analysis has several limitations. First, all included studies are single-center case series or case-control designs without RCTs, introducing inherent selection bias. Second, the two comparative studies did not adjust for key confounding variables such as tumor size, PRETEXT/POST-TEXT stage, and anatomical location, which are primary determinants of surgical approach selection. Third, a comparative survival analysis between LLR and OLR was not feasible due to heterogeneous tumor composition between studies and absence of histology-stratified survival data. Fourth, heterogeneity in outcome definitions across studies and the small number of included studies (9) limited statistical power and precluded predefined subgroup analyses by age, tumor location, and other factors. Fifth, inclusion of hybrid techniques introduces methodological heterogeneity, although sensitivity analysis confirmed stable pooled estimates, this technical heterogeneity remains a methodological concern. Finally, language and publication bias cannot be excluded.

Pediatric liver tumor rarity inherently limits sample sizes and complicates research. Additionally, the proportionally larger tumor burden relative to smaller abdominal cavities in children presents unique technical challenges compared to adults. These limitations necessitate cautious interpretation and extrapolation. Future large-scale, multicenter studies and ideally RCTs are needed to validate these findings and provide more precise evidence for clinical decision-making.

## Supplementary material

Supplementary Figure S1 **(A)** Tumor size, **(B)** Operative time, **(C)** Intraoperative blood loss, **(D)** Proportion of blood transfusion, **(E)** Hospital stay, **(F)** All complications, **(G)** Bile leakage, and **(H)** Hepatoblastoma recurrence. Leave-one-out sensitivity analysis for all pooled clinical characteristics. Each row represents the pooled estimate obtained after sequentially excluding one study at a time. Each blue square represents the pooled mean or proportion from the sensitivity analysis iteration, with horizontal lines indicating 95% confidence intervals (CIs). The size of the square reflects the precision of the estimate. The diamond at the bottom of each panel shows the overall pooled mean or proportion and its 95% CI. Heterogeneity parameters (Tau^2^, Tau, and I^2^) are displayed to show how individual studies influence between-study heterogeneity.
