# Supplementary material for: Laparoscopic liver resection for pediatric liver tumors: a systematic review and meta-analysis
Source: Front Pediatr. 2026 Jul 24;14:1756373. doi: 10.3389/fped.2026.1756373 (PMC13447245; doi:10.3389/fped.2026.1756373)
Supplement: Supplementary file 1 [file Supplementaryfile1.docx]

# 20241212 Searching Strategy

# Pubmed

| Search number | Query | Results |
| --- | --- | --- |
| #1 | ((((("Pediatrics"[Mesh]) OR "Infant, Newborn"[Mesh]) OR "Infant"[Mesh]) OR "Child, Preschool"[Mesh]) OR "Child"[Mesh]) OR "Adolescent"[Mesh] | 4,136,584 |
| #2 | (((((((((((pediatric*[Title/Abstract]) OR (pediatry[Title/Abstract])) OR (paediatric*[Title/Abstract])) OR (newborn*[Title/Abstract])) OR (neonat*[Title/Abstract])) OR (Infan*[Title/Abstract])) OR (child[Title/Abstract])) OR (children*[Title/Abstract])) OR (juvenil*[Title/Abstract])) OR (adolescen*[Title/Abstract])) OR (teen*[Title/Abstract])) OR (youth*[Title/Abstract]) | 2,831,655 |
| #3 | #1 OR #2 | 4,958,871 |
| #4 | ("Liver Neoplasms"[Mesh]) OR "Hepatoblastoma"[Mesh] | 204,576 |
| #5 | ((((((liver neoplasms[Title/Abstract]) OR (liver neoplasm[Title/Abstract])) OR (liver tumor[Title/Abstract])) OR (liver cancer[Title/Abstract])) OR (hepatocellular cancer[Title/Abstract])) OR (hepatic cancer[Title/Abstract])) OR (Hepatoblastoma*[Title/Abstract]) | 50,902 |
| #6 | #4 OR #5 | 223,621 |
| #7 | "Hepatectomy"[Mesh] | 35,920 |
| #8 | ((((hepatectom*[Title/Abstract]) OR (liver resection[Title/Abstract])) OR (hepatic resection[Title/Abstract])) OR (segmentectomy[Title/Abstract])) OR (sectionectomy[Title/Abstract]) | 46,472 |
| #9 | #7 OR #8 | 57,458 |
| #10 | ("Laparoscopes"[Mesh]) OR "Minimally Invasive Surgical Procedures"[Mesh] | 57,394 |
| #11 | (Laparoscop*[Title/Abstract]) OR (Minimally Invasive Surgical Procedures[Title/Abstract]) | 165,645 |
| #12 | #10 OR #11 | 689,213 |
| #13 | #3 AND #6 AND #9 AND #12 | 232 |

# Embase

| Search number | Query | Results |
| --- | --- | --- |
| #1 | 'pediatrics'/exp/mj OR 'newborn'/exp/mj OR 'infant'/exp/mj OR 'preschool child'/exp/mj OR 'child'/exp/mj OR 'adolescent'/exp/mj | 328,188 |
| #2 | pediatric*:ab,ti OR pediatry:ab,ti OR paediatric*:ab,ti OR 'infant, newborn':ab,ti OR newborn*:ab,ti OR neonat*:ab,ti OR infan*:ab,ti OR 'child, preschool':ab,ti OR child:ab,ti OR children*:ab,ti OR adolescent:ab,ti OR juvenil*:ab,ti OR adolescen*:ab,ti OR teen*:ab,ti OR youth*:ab,ti | 3,638,189 |
| #3 | #1 OR #2 | 3,709,746 |
| #4 | 'liver tumor'/exp/mj OR 'hepatoblastoma'/exp/mj | 228,255 |
| #5 | 'liver neoplasms':ab,ti OR 'liver neoplasm':ab,ti OR hepatoblastoma*:ab,ti OR 'liver tumor':ab,ti OR 'liver cancer':ab,ti OR 'hepatocellular cancer':ab,ti OR 'hepatic cancer':ab,ti | 63,914 |
| #6 | #4 OR #5 | 253,168 |
| #7 | 'hepatectomy'/exp/mj OR 'segmentectomy'/exp/mj OR 'sectionectomy'/exp/mj | 32,116 |
| #8 | hepatectoma*:ab,ti OR 'liver resection':ab,ti OR 'hepatic resection':ab,ti OR segmentectomy:ab,ti OR sectionectomy:ab,ti | 35,773 |
| #9 | #7 OR #8 | 52,178 |
| #10 | 'laparoscope'/exp/mj OR 'minimally invasive surgery'/exp/mj | 24,147 |
| #11 | laparoscop*:ab,ti OR 'minimally invasive surgical procedures':ab,ti | 264,120 |
| #12 | #10 OR #11 | 283,177 |
| #13 | #3 AND #6 AND #9 AND #12 | 262 |

# Web of Science

| Search number | Query | Results |
| --- | --- | --- |
| #1 | AB=(pediatric* OR pediatry OR paediatric* OR “infant, newborn” OR newborn* OR neonat* OR infan* OR “Child, Preschool” OR child OR children* OR adolescent OR juvenil* OR adolescen* OR teen* OR youth* ) | 4,213,861 |
| #2 | TI=(pediatric* OR pediatry OR paediatric* OR “infant, newborn” OR newborn* OR neonat* OR infan* OR “Child, Preschool” OR child OR children* OR adolescent OR juvenil* OR adolescen* OR teen* OR youth* ) | 3,354,458 |
| #3 | #1 OR #2 | 5,552,332 |
| #4 | AB=(liver neoplasms OR liver neoplasm OR hepatoblastoma* OR liver tumor OR liver cancer OR hepatocellular cancer OR hepatic cancer ) | 370,043 |
| #5 | TI=(liver neoplasms OR liver neoplasm OR hepatoblastoma* OR liver tumor OR liver cancer OR hepatocellular cancer OR hepatic cancer ) | 81,635 |
| #6 | #4 OR #5 | 394,975 |
| #7 | AB=(hepatectoma* OR liver resection OR hepatic resection OR segmentectomy OR sectionectomy) | 57,143 |
| #8 | TI=(hepatectoma* OR liver resection OR hepatic resection OR segmentectomy OR sectionectomy) | 17,953 |
| #9 | #7 OR #8 | 63,379 |
| #10 | AB=(Laparoscop* OR Minimally Invasive Surgical Procedures) | 222,149 |
| #11 | TI=(Laparoscop* OR Minimally Invasive Surgical Procedures) | 163,217 |
| #12 | #10 OR #11 | 274,357 |
| #13 | #3 AND #6 AND #9 AND #12 | 170 |

# Cochrane Library

| Search number | Query | Results |
| --- | --- | --- |
| #1 | MeSH descriptor: [Pediatrics] explode all trees | 1059 |
| #2 | MeSH descriptor: [Infant, Newborn] explode all trees | 24573 |
| #3 | MeSH descriptor: [Infant] explode all trees | 47027 |
| #4 | MeSH descriptor: [Child, Preschool] explode all trees | 40382 |
| #5 | MeSH descriptor: [Child] explode all trees | 83722 |
| #6 | MeSH descriptor: [Adolescent] explode all trees | 139416 |
| #7 | (pediatric* OR pediatry OR paediatric* OR 'infant, newborn' OR newborn* OR neonat* OR infan* OR 'child, preschool' OR child OR children* OR adolescent OR juvenil* OR adolescen* OR teen* OR youth*):ti,ab,kw | 375820 |
| #8 | #1 OR #2 OR #3 OR #4 OR #5 OR #6 OR #7 | 375837 |
| #9 | MeSH descriptor: [Liver Neoplasms] explode all trees | 4576 |
| #10 | MeSH descriptor: [Hepatoblastoma] explode all trees | 29 |
| #11 | (liver neoplasms OR liver neoplasm OR hepatoblastoma* OR liver tumor OR liver cancer OR hepatocellular cancer OR hepatic cancer):ti,ab,kw | 21697 |
| #12 | #9 OR #10 OR #11 | 22178 |
| #13 | MeSH descriptor: [Hepatectomy] explode all trees | 977 |
| #14 | (hepatectoma* OR liver resection OR hepatic resection OR segmentectomy OR sectionectomy):ti,ab,kw | 5748 |
| #15 | #13 OR #14 | 5971 |
| #16 | MeSH descriptor: [Laparoscopes] explode all trees | 146 |
| #17 | MeSH descriptor: [Minimally Invasive Surgical Procedures] explode all trees | 44890 |
| #18 | (Laparoscop* OR Minimally Invasive Surgical Procedures):ti,ab,kw | 32823 |
| #19 | #16 OR #17 OR #18 | 66886 |
| #20 | #8 AND #12 AND #15 AND #19 | 48 |

# CNKI

Query:

（主题：肝肿瘤）OR（篇关摘：肝肿瘤 + 肝脏肿瘤 + 肝母细胞瘤 + 原发性肝癌 + 肝细胞癌 + 肝癌（模糊））AND（主题：肝切除术）OR（篇关摘：肝切除术 + 手术切除 + 肝切除 + 肝癌切除术 + 肝肿瘤切除术 + 手术治疗 + 外科手术 + 肿瘤切除 + 肝脏外科）AND（篇关摘：腹腔镜 + 微创（模糊））AND（全文：新生儿 + 儿童 + 青少年 + 儿科 + 小儿（模糊））

Results:

208

# WanFang

Query:

（中英文扩展&主题词扩展）： 全部:(新生儿 OR 儿童 OR 青少年 OR 儿科 OR 小儿) and 题名或关键词:(肝肿瘤 OR 肝脏肿瘤 OR 肝母细胞瘤 OR 原发性肝癌 OR 肝细胞癌 OR 肝癌) and 题名或关键词:(肝切除术 OR 手术切除 OR 肝切除 OR 肝癌切除术 OR 肝肿瘤切除术 OR 手术治疗 OR 外科手术 OR 肿瘤切除 OR 肝脏外科) and 题名或关键词:(腹腔镜 OR 微创)

Results:

55

# VIP database

Query:

(((((((任意字段=新生儿 OR 任意字段=儿童) OR 任意字段=青少年) OR 任意字段=儿科) OR 任意字段=小儿) AND (((((题名或关键词=肝肿瘤 OR 题名或关键词=肝脏肿瘤) OR 题名或关键词=肝母细胞瘤) OR 题名或关键词=原发性肝癌) OR 题名或关键词=肝细胞癌) OR 题名或关键词=肝癌)) AND ((((((((题名或关键词=肝切除术 OR 题名或关键词=手术切除) OR 题名或关键词=肝切除) OR 题名或关键词=肝癌切除术) OR 题名或关键词=肝肿瘤切除术) OR 题名或关键词=手术治疗) OR 题名或关键词=外科手术) OR 题名或关键词=肿瘤切除) OR 题名或关键词=肝脏外科)) AND (题名或关键词=腹腔镜 OR 题名或关键词=微创)) Results: 15

# Inclusion criteria:

① patients aged ≤18 years;

② diagnosed with benign or malignant liver tumors;

③ treated with laparoscopic liver resection;

④ with available data on operative time, intraoperative blood loss, postoperative complications, and length of hospital stay;

⑤ for articles from the same research center with overlapping cases, if non‑overlapping data could be obtained, they were included; otherwise, priority was given to studies with a larger number of cases, more recent publication, more complete data, and higher quality.

# Exclusion criteria:

① articles not published in English or Chinese;

② reviews, conference abstracts, comments, case reports, dissertations, clinical trial registration records, etc.;

③ cellular or animal experiments;

④ articles for which the full text was not available.
