# Supplementary material for: Laparoscopic liver resection for pediatric liver tumors: a systematic review and meta-analysis
Source: Front Pediatr. 2026 Jul 24;14:1756373. doi: 10.3389/fped.2026.1756373 (PMC13447245; doi:10.3389/fped.2026.1756373)
Supplement: Supplementary file 2 [file Table1.docx]

**Table S1 Clinical characteristics and outcomes of included comparative studies.**

| **Author** |  | **LLR** | **OLR** |
| --- | --- | --- | --- |
| **Zheng B, 2019** | Patient (n) | 17 | 26 |
|  | Tumor size (cm) | 7.0±4.6 | 8.8±3.3 |
|  | PRE/POST-TEXT (n) | I/II/III/IV (2/5/9/1)^a^ | I/II/III/IV (3/8/12/3)^a^ |
|  | Resection Type (n) | Right inferior hepatic segment (4); Right posterosuperior hepatic segment (1); Left lateral section (3); Left hemihepatectomy (2); Left hemihepatectomy with right hepatic tumor resection (1); Left trisectionectomy (2); Right hemihepatectomy (3); Right hemihepatectomy with left hepatic tumor resection (1); Right trisectionectomy (0) | Right inferior hepatic segment (5); Right posterosuperior hepatic segment (2); Left lateral section (3); Left hemihepatectomy (4); Left hemihepatectomy with right hepatic tumor resection (1); Left trisectionectomy (3); Right hemihepatectomy (5); Right hemihepatectomy with left hepatic tumor resection (2); Right trisectionectomy (1) |
|  | Outcome (n) | HB: Relapse (3) | HB: Relapse (3); lung metastasis (3) |
| **Wang J, 2020** | Patient (n) | 21 | 9 |
|  | Tumor size (cm) | 7.2±2.0 (4-10) | 7.5±2.2 (4.4-10) |
|  | PRE/POST-TEXT (n) | NR | NR |
|  | Resection Type (n) | Left hepatectomy (5); left lateral hepatic lobectomy (6); nonanatomic hepatectomy (10) | Left hepatectomy (3); left lateral hepatic lobectomy (2); nonanatomic hepatectomy (4) |
|  | Outcome (n) | No recurrence | No recurrence |

^a^PRETEXT stage; LLR: laparoscopic liver resection; OLR:open liver resection; HB, hepatoblastoma. NR, Not Reported.
