# Supplementary material for: Laparoscopic liver resection for pediatric liver tumors: a systematic review and meta-analysis
Source: Front Pediatr. 2026 Jul 24;14:1756373. doi: 10.3389/fped.2026.1756373 (PMC13447245; doi:10.3389/fped.2026.1756373)
Supplement: Supplementary file 3 [file Table2.docx]

**Table S2.**

**Leave-One-Out Sensitivity Analysis for All Pooled characteristics and outcomes.**

| **Outcome** | **All Studies (n)** | **Pooled Estimate [95% CI]** | **Range (Exclusion)** | **Max Variation** |
| --- | --- | --- | --- | --- |
| (A) Tumor Size (cm) | 8 | 5.69 [4.85–6.53] | 5.24–5.86 | ±0.08 cm (1.4%) |
| (B) Operative Time (min) | 9 | 208.77 [153.74–263.80] | 194.95–223.03 | ±14.3 min (6.8%) |
| (C) Blood Loss (mL) | 7 | 88.80 [31.33–146.27] | 65.75–99.39 | ±10.6 mL (12%) |
| (D) Blood Transfusion Proportion | 5 | 31.6% [15.6%–53.7%] | 25.6%–45.1% | ±13.5% |
| (E) Hospital Stay (days) | 7 | 6.80 [4.03–9.57] | 5.70–7.28 | ±0.5 days (7%) |
| (F) All Complications Proportion | 9 | 15.8% [10.1%–23.9%] | 13.7%–16.9% | ±1.1% |
| (G) Bile Leakage Proportion | 9 | 10.6% [5.8%–18.6%] | 8.6%–11.9% | ±1.3% |
| (H) Recurrence (HB) Proportion | 7 | 9.4% [4.5%–18.5%] | 6.0%–10.5% | ±1.1% |

Values are presented as pooled estimates with 95% confidence intervals [CI]. CI, confidence interval; HB, hepatoblastoma. Leave-one-out sensitivity analysis was performed by sequentially excluding each included study and recalculating the pooled estimate. Reported are the range of pooled estimates across all exclusion scenarios and maximum absolute/relative variation. All confidence intervals remained overlapping across sensitivity scenarios, and the direction of estimates was consistent, indicating robustness of findings despite heterogeneity.

**Table S2-A. Tumor Size**

| **Excluded_study** | **Pooled_mean** | **CI_lower** | **CI_upper** |
| --- | --- | --- | --- |
| None (all studies) | 5.69 | 4.85 | 6.53 |
| Veenstra MA, 2016^a^ | 5.72 | 4.72 | 6.72 |
| Zheng B, 2019 | 5.58 | 4.68 | 6.48 |
| Kwon H, 2019^a^ | 5.83 | 4.86 | 6.79 |
| Wang J, 2020 | 5.24 | 4.62 | 5.85 |
| Murawski M, 2021^a^ | 5.81 | 4.89 | 6.74 |
| Li Q, 2021^a^ | 5.86 | 4.95 | 6.77 |
| Zheng B, 2022 | 5.63 | 4.65 | 6.60 |
| Qiu R, 2022^a^ | 5.69 | 4.70 | 6.68 |
| Veenstra MA & Wang J (both) | 5.19 | 4.42 | 5.95 |

*^a^underwent median-to-mean conversion.*

**Table S2-B. Operative Time**

| **Excluded_study** | **Pooled_mean** | **CI_lower** | **CI_upper** |
| --- | --- | --- | --- |
| None (all studies) | 208.77 | 153.74 | 263.80 |
| Veenstra MA, 2016^a^ | 215.53 | 153.67 | 277.39 |
| Zheng B, 2019 | 204.22 | 141.09 | 267.35 |
| Kwon H, 2019 | 208.00 | 143.77 | 272.23 |
| Wang J, 2020 | 212.74 | 148.98 | 276.50 |
| Murawski M, 2021 | 212.88 | 149.69 | 276.06 |
| Li Q, 2021 | 223.03 | 177.03 | 269.02 |
| Zhou H, 2021 | 203.15 | 140.62 | 265.68 |
| Zheng B, 2022 | 205.91 | 142.07 | 269.76 |
| Qiu R, 2022 | 194.95 | 148.09 | 241.81 |
| Veenstra MA & Wang J (both) | 221.06 | 147.95 | 294.18 |

*^a^underwent median-to-mean conversion.*

**Table S2-C. Blood Loss**

| **Excluded_study** | **Pooled_mean** | **CI_lower** | **CI_upper** |
| --- | --- | --- | --- |
| None (all studies) | 88.80 | 31.33 | 146.27 |
| Veenstra MA, 2016^a^ | 98.96 | 24.03 | 173.89 |
| Zheng B, 2019 | 96.39 | 17.09 | 175.69 |
| Wang J, 2020 | 65.75 | 37.62 | 93.88 |
| Li Q, 2021^a^ | 95.40 | 15.79 | 175.00 |
| Zhou H, 2021^a^ | 99.39 | 26.18 | 172.60 |
| Zheng B, 2022^a^ | 98.59 | 22.15 | 175.04 |
| Qiu R, 2022 | 77.41 | 37.55 | 117.28 |
| Veenstra MA & Wang J (both) | 69.3 | 28.53 | 110.08 |

*^a^underwent median-to-mean conversion.*

**Table S2-D. Proportion of Blood Transfusion**

| **Excluded_study** | **Pooled_rate** | **CI_lower** | **CI_upper** |
| --- | --- | --- | --- |
| None (all studies) | 0.32 | 0.16 | 0.54 |
| Veenstra MA, 2016 | 0.45 | 0.31 | 0.60 |
| Zheng B, 2019 | 0.26 | 0.11 | 0.50 |
| Kwon H, 2019 | 0.27 | 0.10 | 0.53 |
| Murawski M, 2021 | 0.31 | 0.12 | 0.58 |
| Li Q, 2021 | 0.35 | 0.17 | 0.58 |
| Veenstra MA & Wang J (both) | 0.45 | 0.31 | 0.60 |

**Table S2-E. Hospital Stay**

| **Excluded_study** | **Pooled_mean** | **CI_lower** | **CI_upper** |
| --- | --- | --- | --- |
| None (all studies) | 6.80 | 4.03 | 9.57 |
| Veenstra MA, 2016^a^ | 7.21 | 3.99 | 10.44 |
| Zheng B, 2019 | 6.72 | 3.28 | 10.16 |
| Kwon H, 2019 | 6.52 | 3.25 | 9.78 |
| Wang J, 2020 | 6.91 | 3.47 | 10.35 |
| Murawski M, 2021 | 7.14 | 3.84 | 10.43 |
| Li Q, 2021 | 7.28 | 4.14 | 10.42 |
| Zhou H, 2021 | 5.70 | 3.90 | 7.50 |
| Veenstra MA & Wang J (both) | 7.43 | 3.22 | 11.65 |

*^a^underwent median-to-mean conversion.*

**Table S2-F. All Complications**

| **Excluded_study** | **Pooled_rate** | **CI_lower** | **CI_upper** |
| --- | --- | --- | --- |
| None (all studies) | 0.16 | 0.10 | 0.24 |
| Veenstra MA, 2016 | 0.17 | 0.10 | 0.27 |
| Zheng B, 2019 | 0.14 | 0.09 | 0.23 |
| Kwon H, 2019 | 0.17 | 0.11 | 0.25 |
| Wang J, 2020 | 0.17 | 0.11 | 0.25 |
| Murawski M, 2021 | 0.16 | 0.10 | 0.25 |
| Li Q, 2021 | 0.16 | 0.10 | 0.25 |
| Zhou H, 2021 | 0.14 | 0.08 | 0.22 |
| Zheng B, 2022 | 0.17 | 0.11 | 0.26 |
| Qiu R, 2022 | 0.15 | 0.09 | 0.23 |
| Veenstra MA & Wang J (both) | 0.18 | 0.10 | 0.30 |

**Table S2-G. Bile Leakage**

| **Excluded_study** | **Pooled_rate** | **CI_lower** | **CI_upper** |
| --- | --- | --- | --- |
| None (all studies) | 0.11 | 0.06 | 0.19 |
| Veenstra MA, 2016 | 0.12 | 0.06 | 0.21 |
| Zheng B, 2019 | 0.09 | 0.04 | 0.17 |
| Kwon H, 2019 | 0.12 | 0.06 | 0.20 |
| Wang J, 2020 | 0.12 | 0.06 | 0.20 |
| Murawski M, 2021 | 0.11 | 0.06 | 0.19 |
| Li Q, 2021 | 0.11 | 0.06 | 0.19 |
| Zhou H, 2021 | 0.10 | 0.05 | 0.19 |
| Zheng B, 2022 | 0.11 | 0.06 | 0.20 |
| Qiu R, 2022 | 0.09 | 0.04 | 0.16 |
| Veenstra MA & Wang J (both) | 0.13 | 0.07 | 0.23 |

**Table S2-H. Hepatoblastoma Recurrence**

| **Excluded_study** | **Pooled_rate** | **CI_lower** | **CI_upper** |
| --- | --- | --- | --- |
| None (all studies) | 0.09 | 0.04 | 0.18 |
| Veenstra MA, 2016 | 0.10 | 0.05 | 0.21 |
| Zheng B, 2019 | 0.06 | 0.02 | 0.15 |
| Kwon H, 2019 | 0.10 | 0.05 | 0.20 |
| Wang J, 2020 | 0.10 | 0.05 | 0.20 |
| Li Q, 2021 | 0.09 | 0.04 | 0.19 |
| Zheng B, 2022 | 0.10 | 0.04 | 0.20 |
| Qiu R, 2022 | 0.10 | 0.04 | 0.20 |
| Veenstra MA & Wang J (both) | 0.11 | 0.05 | 0.23 |
